# Evaluation of Cyprinid Herpesvirus 2 Latency and Reactivation in *Carassius gibel*

**DOI:** 10.3390/microorganisms8030445

**Published:** 2020-03-21

**Authors:** Wenjun Chai, Lin Qi, Yujun Zhang, Mingming Hong, Ling Jin, Lijuan Li, Junfa Yuan

**Affiliations:** 1 Department of Aquatic Animal Medicine, College of Fisheries, Huazhong Agricultural University, Wuhan 430070, China; wenjunchai1994@163.com (W.C.); qilin0401@webmail.hzau.edu.cn (L.Q.); ZYJ415@webmail.hzau.edu.cn (Y.Z.); hongmm727@126.com (M.H.); Lilijuan@mail.hzau.edu.cn (L.L.); 2 Department of Biomedical Science, Carlson College of Veterinary Medicine, Oregon State University, Corvallis, OR 97330, USA; ling.jin@oregonstate.edu; 3 Hubei Engineering Research Center for Aquatic Animal Diseases Control and Prevention, Wuhan 430070, China

**Keywords:** Cyprinid herpesvirus 2, latency, cell line, gibel carp, reactivation

## Abstract

Cyprinid herpesvirus 2 (CyHV-2, species *Cyprinid herpesvirus 2*) causes severe mortality in ornamental goldfish, crucian carp (*Carassius auratus*), and gibel carp (*Carassius gibelio*). It has been shown that the genomic DNA of CyHV-2 could be detected in subclinical fish, which implied that CyHV-2 could establish persistent infection. In this study, the latency of CyHV-2 was investigated in the survival fish after primary infection. CyHV-2 genomic DNA was detected in multiple tissues of acute infection samples; however, detection of CyHV-2 DNA was significantly reduced in fish recovered from the primary infection on day 300 postinfection. No active viral gene transcription, such as DNA polymerase and *ORF99*, was detected in recovered fish. Following temperature stress, an increase of CyHV-2 DNA copy numbers and gene transcription were observed in tissues examined, which suggests that CyHV-2 was reactivated under stress. In addition, a cell line (GCBLat1) derived from the brain tissue from CyHV-2-exposed fish harbored CyHV-2 genome but did not produce infectious virions under normal culture conditions. However, CyHV-2 replication and viral gene transcription occurred when GCBLat1 cells were treated with trichostatin A (TSA) or phorbol 12-myristate 13-acetate (TPA). It suggests CyHV-2 can remain latent in vitro and can reactivate under stress condition.

## 1. Introduction

Cyprinid herpesvirus 2 (CyHV-2, species *Cyprinid herpesvirus 2*), also known as herpesviral hematopoietic necrosis virus of goldfish (*Carassius auratus*), causes high mortality in the farmed ornamental goldfish crucian carp (*Carassius carassius*) and gibel carp (*Carassius gibelio*) [1]. Outbreaks of CyHV-2 in wild gibel carp were also reported in natural water reservoirs in Hungary, Czech Republic, and Italy [2,3]. Acute infection of CyHV-2 often occurs at a water temperature range of 15–25 °C and causes necrosis of gill and hematopoietic organs [4]. It has been reported that the genomic DNA of CyHV-2 could be detected in subclinical fish [5,6]. In addition, injection of a kidney homogenate from those subclinical fish can cause acute CyHV-2 infection and mortality in the naïve fish [7]. These studies implied that CyHV-2 could establish a persistent infection.

CyHV-2 is a member of the genus of *Cyprinivirus*, in the family *Alloherpesviridae* within the order *Herpesvirales* [8]. One of the unique features of herpesviruses is latency and reactivation [9]. Many members of *Herpesviridae*, such as human herpes simplex virus (HSV), a member of the subfamily Alphaherpesvirinae, can establish latency primarily in sensory neurons of the peripheral nervous system [10]. Epstein–Barr virus (EBV), a member of the Gammaherpesvirinae, can become latent in memory B cells, monocytes, or lymphocytes [11]. Among the viruses in the family *Alloherpesviridae*, channel catfish virus (CCV, species *Ictalurid herpesvirus 1*) and koi herpesvirus (KHV, species *Cyprinid herpesvirus 3*) were reported to become latent in lymphocytes following an acute infection [12,13]. However, CyHV-2 latency has not been characterized yet. During the latent state, the carriers do not produce any infectious virus and are subclinical [14,15]. However, reactivation from latency may occur under stressful conditions; the carriers will produce infectious viruses and infect naïve fish [16]. Therefore, demonstrating CyHV-2 latency in recovered fish is important for the development of control strategies against CyHV-2 infection.

In this study, the latency of CyHV-2 was investigated in the recovered fish following acute infection. CyHV-2 reactivation from latency in the recovered fish was investigated through temperature stress. In addition, a cell line from the latently infected fish was shown to harbor CyHV-2 genome without the production of infectious virions under normal culture conditions. Furthermore, CyHV-2 latent genome in the cells can be reactivated by treatment of trichostatin A (TSA) or phorbol 12-myristate 13-acetate (TPA).

## 2. Materials and Methods

### 2.1. Virus, Fish, and Experimental Infection

The CyHV-2 SY-C1 isolate was used in this study [17]. Healthy gibel carp were obtained from the South Lake Science Station, Huazhong Agricultural University (HZAU), Wuhan. These fish were kept in a quarantine tank at 23–25 °C with a natural photoperiod for 14 days. All fish used in this study were housed and cared at HZAU in accordance with Institutional Animal Care and Use Committee guidelines that was approved by the Scientific Ethic Committee of Huazhong Agricultural University (HZAUFI-2018-016).

A total of 100 fish were divided into two groups: one was used for CyHV-2 infection and the other half served as control. Fish were infected intraperitoneally with 0.1 mL virus stock containing 100,000 copy numbers of CyHV-2 (0.1 × LD_50,_) as descripted previously [17]. Briefly, 1 g of kidney tissue from CyHV-2-infected gibel carp were pooled and homogenized in 10 mL of TN buffer (50 mmol/L Tris–HCl, 100 mmol/L NaCl, pH 7.4), followed by centrifugation at 5000 *g* for 10 min at 4 °C. Subsequently, the supernatant was filtered through a 0.45-µm acetate cellulose filter and subjected for virus quantitate by qPCR. All fish in the control group were injected intraperitoneally with the same volume of TN buffer. During the period of infection, all fish were kept at 23–25 °C with a natural photoperiod. The gills, liver, spleen, trunk kidney, intestine, heart, brain, and caudal fin were sampled from 4 moribund fish on five days postinfection, and four recovered fish on 300 days postinfection.

### 2.2. Temperature Stress

To determine whether CyHV-2 latency can be reactivated, 20 surviving fish at 300 days postinfection were subjected to temperature stress as described previously [12,16]. The tank water temperature was increased from 14 °C to 25 °C at a rate of 1 °C per day and was then held constant for 5 days at 25 °C before being dropped back to 14 °C at a rate of 1 °C per day. All experimental fish were fed twice daily and maintained with a natural photoperiod. The dead fish were necropsied, and tissue samples were collected, including gills, liver, spleen, trunk kidney, intestine, heart, brain, and caudal fins.

### 2.3. Detection of CyHV-2 Genomic DNA

Total DNA from each tissue was extracted using a Tissue DNA Purification Kit according to the manufacturer’s instructions (CWBio, Beijing, China). The extracted DNA was screened by PCR with CyHV-2 specific primers for viral DNA detection as described before [17,18]. The PCR product was visualized in 1.5% agarose gel electrophoresis with Gel-Red (Biosharp, Hefei, China) staining.

### 2.4. Quantitative PCR Assay

Quantitative PCR (qPCR) was performed with primers specific to the DNA polymerase gene (*DNA pol*) of CyHV-2. The reaction in 20 µL reactions contained 10 µL of SYBR^®^ Premix Ex Taq TM II (2×) and 0.5 µL of qpol-F and qpol-R primers each. The reactions were run at 95 °C for 5 min and at 40 cycles of 95 °C for 5 s, 58 °C for 30 s, 72 °C for 30 s, and then 72 °C for 10 min. The primer pairs used in qPCR are listed in Table 1. Viral genome copies were calculated according to the standard curve, which was constructed based on the series of diluted plasmids containing the DNA polymerase gene, and the results were expressed with copies per µg total DNA (copies/µg DNA).

### 2.5. RNA Extraction, Synthesis of cDNA, and PCR Analysis

The total RNA was extracted from each sample using TRIzol reagent (Invitrogen, Carlsbad, CA, USA) according to the manufacturer’s protocol. To avoid viral genomic DNA containment, recovered RNA sample was treated with RQ1 RNase free DNase (Promega, Madison, WI, USA) at 37 °C for 30 min. Two microgram (µg) of treated total RNA was reverse-transcribed to cDNA using M-MLV reverse transcriptase (RTase) with random primers according to the manufacturer’s protocol. To ensure RTase worked properly, an equal amount of RNA sample was used in the RT reaction without including the RTase. The cDNA was subsequently amplified in PCR using primers specific for viral *ORF99* and *DNA pol*. The amplification of beta-actin gene (*β-actin*) was performed as an internal control to ensure that comparable levels of input RNA were used in RT.

### 2.6. Primary Cell Culture and Subculture

The entire brain from CyHV-2 latently infected fish was collected and washed twice in Medium 199 containing streptomycin (100 µg/mL) and penicillin (100 U/mL). The washed brain was minced with scissors into small pieces and digested into a single cell suspension with 0.25% trypsin-versene solution (Sigma-Aldrich, St. Louis, MO, USA) at room temperature for 15 min. The digested cells were then placed in M199 medium containing 10% heat-inactivated fetal bovine serum (FBS, Invitrogen, Carlsbad, CA, USA) and filtered with a 70-µm steel mesh. The filtered cell suspensions were centrifuged at 200× *g* for 5 min and resuspended in M199 medium supplemented with 20% FBS containing streptomycin and penicillin antibiotics and cultured in 25 cm^2^ tissue culture flasks (Corning, NY, USA) at 28 °C with 5% CO_2_. Approximately 50% of the medium was replaced with fresh cell culture medium every 3 days. The confluent monolayer was split at a ratio of 1:2 every 6–8 days. After 15 subcultures, the cells were cultured in M199 medium with 10% FBS.

### 2.7. Cell Characterization

The primary brain cell culture was named the GCBLat1 cell line. GCBLat1 cells at passage 30 were seeded onto 12-well plates in 1 mL of M199 medium containing 10% FBS at an initial density of 3 × 10^5^ cells per well. On day 1 to 4, trypsinized cells were counted by a haemocytometer. The average cell number of three wells at each time point was used to plot a cell growth curve.

Chromosome numbers of GCBLat1 cells were analyzed from GCBLat1 cells at the 36th passage as previous descriptions [19,20]. Briefly, cells were seeded in 25 cm^2^ until they reached 80% confluence and then incubated with colchicine (Sigma-Aldrich, St. Louis, MO, USA) in a final concentration of 0.2 μg/mL for 15 h. The trypsinized cells were pelleted by centrifugation at 180× *g* for 5 min and resuspended in 8 mL of 75 mM KCl for 25 min. The treated cells were then fixed in 3 mL methanol: acetic acid (3:1) for 10 min. The final cell suspension was dropped onto a clean precooled microslide, stained with Giemsa (Sigma-Aldrich, St. Louis, MO, USA) for 15 min at room temperature and then examined under a Leica light microscope.

### 2.8. Reactivation of CyHV-2 In Vitro

Trichostatin A (TSA, T8552) and phorbol 12-myristate 13-acetate (TPA, 79346) were obtained from Sigma-Aldrich and diluted in 100% dimethyl sulfoxide (DMSO) and ethanol, respectively. Cell viability following treatment of TSA or TPA was measured by using the MTT assay. Briefly, GCBLat1 cells at 1 × 10^4^ cells per well in 96-well plates were cultured in M199 supplemented with 10% FBS. After treatment with TSA or TPA, cells were stained with MTT (5 mg/mL) at 28 °C for 4 h. Then, 150 ul DMSO was added to each well to dissolve the formazan crystals after removing the medium. The optical density (OD) was determined at 570 nm using a microplate reader (Infinite 200 PRO, Switzerland). The viability of GCBLat1 cells was expressed as a ratio to the vehicle control (DMSO or ethanol).

To investigate whether latent CyHV-2 could be reactivated, GCBLat1 cells at 1 × 10^5^ cells per well in 12-well plates were cultured in M199 supplemented with 10% FBS and treated with 500 ng/mL TPA or 100 nM TSA for 3–5 days. DMSO was used as vehicle control. Three biological replicates were used for each treatment.

### 2.9. Statistical Analysis

The statistical *p* values were calculated by one-way analysis of variance (ANOVA) with the least significant difference test using Prism software (GraphPad). *p* values of less than 0.05 were considered statistically significant (* *p* < 0.05).

## 3. Results

### 3.1. CyHV-2 Established Latency Following Primary Infection

**Figure 1:**
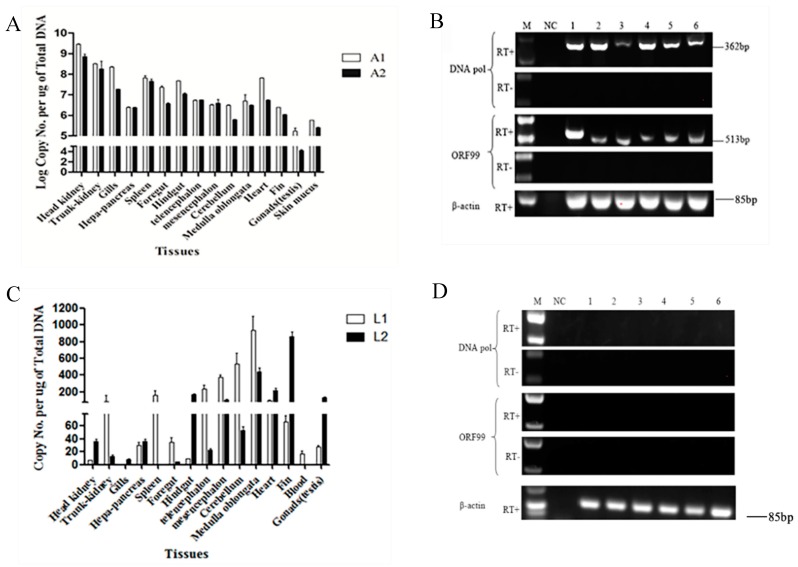
Detection of CyHV-2 DNA and gene transcription from moribund fish or surviving fish after primary infection with CyHV-2: Quantification of the CyHV-2 DNA in tissues from moribund fish (**A**) or surviving fish (**C**) by qPCR. White bars and black bars represent two moribund fish A1 and A2 in panel A and two surviving fish in panel C, respectively. CyHV-2 DNA copies were calculated according to the standard curve based on the series of diluted plasmids containing the DNA polymerase gene and were expressed as copies per µg of total DNA (copies/µg DNA). Data in A and C are presented as the mean of triplicates ± SD of each tested tissue sample from two representative fish. RT-PCR analysis of total RNA harvested from moribund fish (**B**) or surviving fish (**D**) tissues after primary infection with CyHV-2 in the presence (RT+) or absence (RT-) of reverse transcriptase: The amplicons of *DNA pol* (362 bp) and *ORF99* (513 bp) are indicated. Lanes 1 to 6 represent the tissue of head-kidney, gills, midbrain, medulla oblongata, heart, and spleen, respectively. The amplicons of β-actin gene are used as an internal control to ensure that comparable levels of input RNA were used in RT-PCR. One representative experiment of three is shown.

Herpesvirus infections can cause acute infections and diseases in infected individuals. Following the acute infection, herpesviruses become latent in the recovered hosts and remain latent for life within the infected hosts. To investigate whether CyHV-2 can establish latency following a primary infection, a sublethal does of CyHV-2 was injected to 50 fish. About 30% of cumulative mortality was observed between 3 and 14 days postinfection (dpi) with typical clinical signs including gill necrosis and hemorrhage. To measure the viral load during the acute infection, viral DNA was examined in tissues collected from 5 dead fish on day 5 postinfection. As shown in Figure 1A, on average, 10^4^ to 10^9^ of viral DNA per microgram tissue were detected by real-time PCR in multiple tissues in the two tested fish (A1 and A2). Similar results were observed in the other two tested fish, as shown in Figure 1A. By 14 dpi, no mortality and no sick fish were observed in the infected group. No disease and no death were observed in the uninfected group. To further confirm an active infection occurred in those infected fish, transcription of lytic genes, such as *DNA poly* and *ORF99* in CyHV-2 replication was examined in tissues collected from the five dead fish on 5 dpi. As shown in Figure 1B, RT-PCR products at the expected size were amplified from total RNA samples of head-kidney, gills, midbrain, medulla oblongata, heart, and spleen from one of the dead fish. Similar results were observed in tissues collected from the other 3 dead fish (data not shown here). No amplification was observed in RT reactions without the reverse transcriptase (Figure 1B, RT-). No amplification was observed in RT reactions with total RNA isolated from tissues of uninfected fish (data not shown here).

Ten months postinfection, the remaining infected fish all recovered with no clinical signs of the infection. To determine whether CyHV-2 become latent or whether the recovered fish harbored any CyHV-2 viral DNA, various tissues, including head-kidney, gills, midbrain, medulla oblongata, heart, and spleen, were collected from 4 recovered fish and examined by real-time PCR specific to CyHV-2. On average, viral genome copies between 20 to 1000 were detected in multiple tissues from fish that survived from the primary infection (Figure 1C). Similar results were observed in tissues from the other 2 tested fish (data not shown here). To determine whether an active infection was present in these recovered fish, viral gene transcription was examined by RT-PCR. When total RNA samples from these CyHV-2 survival fish were tested for viral gene transcription of *DNA pol* and *ORF99*, which occurs during productive infection, no product was amplified in head-kidney, gills, midbrain, medulla oblongata, heart, and spleen (Figure 1D). Similar results were observed in tissues collected from the other three fish (data not shown here).

### 3.2. CyHV-2 Reactivation under Temperature Stress

**Figure 2:**
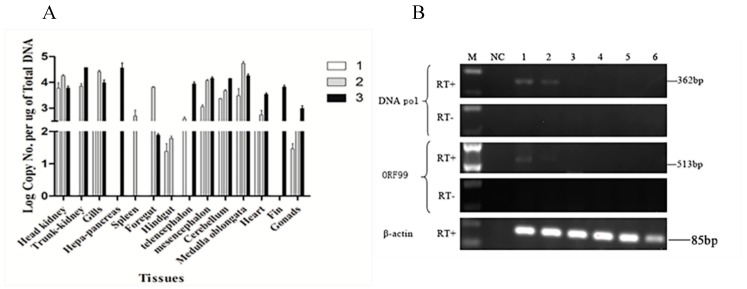
Detection of CyHV-2 DNA and gene transcription in the moribund fish from temperature stress: (**A**) Quantification of the CyHV-2 DNA from three moribund fish suffered temperature stress by qPCR. White (1), gray (2), and black (3) bars respectively represent the different moribund fish. CyHV-2 DNA copies were calculated similarly, as described in Figure 1. The DNA copies shown are the mean of triplicates ± SD. (**B**) RT-PCR analysis of total RNA harvested from moribund fish in the presence (RT+) or absence (RT-) of reverse transcriptase: The amplicons of *DNA pol* (362 bp) and *ORF99* (513 bp) are indicated. Lanes 1 to 6 respectively represent the tissue of the head, kidney, gills, midbrain, medulla oblongata, heart, and spleen, which were sampled from the same fish. The amplicons of β-actin gene are used as an internal control to ensure that comparable levels of input RNA were used in RT-PCR. One representative fish of three is shown.

Since CyHV-2 DNA can be detected in recovered fish, it suggests CyHV-2 is also capable of becoming latent. To determine whether CyHV-2 latency can be reactivated, 20 surviving fish were subjected to temperature stress. A total of four fish were diedwith clinical sign of CyHV-2 infection when the water temperature was increased between 22–24 °C. No mortality was observed when the water temperature was decreased to 14 °C. Tissues from 3 dead fish were collected and tested for CyHV-2 active infection. As shown in Figure 2A, the amount of CyHV-2 DNA copy numbers in most of the tested tissues from those moribund fish went up to 1 × 10^5^ copies/μg, which was about 1000-fold higher than those in latent fish tissues. In agreement with the increase of CyHV-2 DNA copy numbers, the transcription of *DNA pol* and *ORF99* was also detected in several tested tissues including trunk kidney and gills (Figure 2B, lanes 1 and 2). These results indicated that CyHV-2 was reactivated from latency upon temperature stress.

### 3.3. CyHV-2 Latency in the GCBLat1 Cell Line

**Figure 3:**
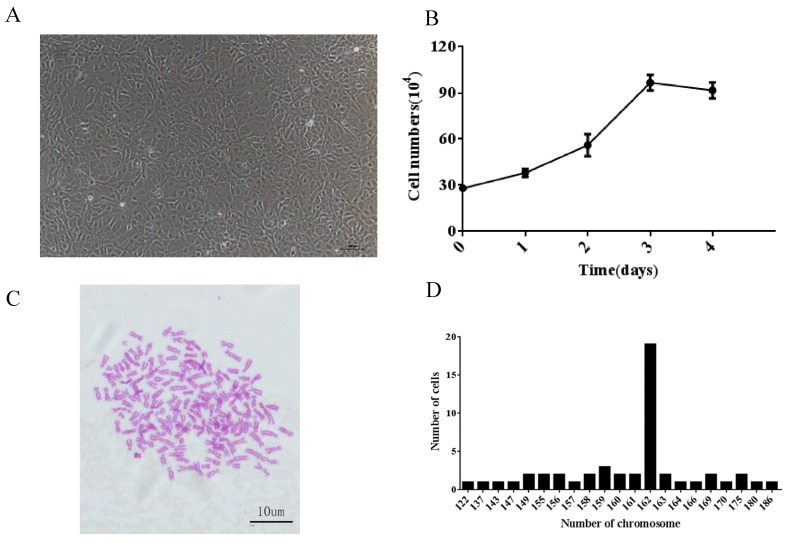
Characteristics of the GCBlat1 cell line: (**A**) Morphology of GCBlat1 cells at passage 80. Scale bars = 100 μm. (**B**) Growth curves of the GCBlat1 cell line at passage 30 at 28 °C within 4 days. Y-axis represents cell numbers of the mean ± standard deviation of three independent experiments. (**C**) Morphological characteristics of chromosomes arrested in metaphase at passage 36. (**D**) Frequency distribution of chromosomes: The *x*-axis represents the number of chromosomes; the *y*-axis represents the number of cells analyzed.

The number of CyHV-2 genome harbored in brain tissues is relatively higher in the latently infected fish (Figure 1C). Therefore, brain tissues from those survival fish were cultured as primary culture. Initially, it took eight days for the primary cultures to grow confluence at 28 °C in M199 medium with 20% FBS. The primary cells were subcultured in M199 medium with 20% FBS every 5–8 days for 10 passages and then subcultured every 3–4 days. To date, the GCBLat1 cells have been subcultured for more than 80 passages over one year. Morphologically, the GCBLat1 cells at 80 passages had epithelial-like cells predominantly (Figure 3A).

The growth rate of GCBLat1 cells was evaluated at passage 30. At 28 °C, GCBLat1 cells proliferated slowly during the first 24 h and then grew faster during 48 and 72 h in M199 medium with 10% FBS. The doubling time of the cell population was 48 h (Figure 3B).

GCBLat1 cells at passages 36 were used to determine their chromosome number. Chromosomal counts from 50 random metaphase plates were used for each cell (Figure 3C). Chromosome numbers ranged from 122 to186, with a distinct peak at 162 (Figure 3D).

**Figure 4:**
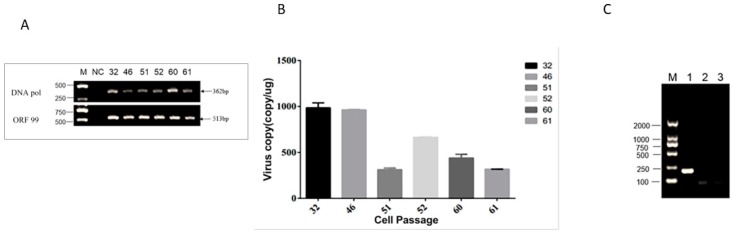
Examination of CyHV-2 DNA and gene transcription in the GCBlat1 cell line: (**A**) PCR analysis of the CyHV-2 DNA from GCBlat1 cells at indicated passages. Amplicons of *DNA pol* (362 bp) and *ORF 99* (513 bp) are indicated. (**B**) qPCR analysis of the CyHV-2 DNA in GCBlat1 cells at indicated passages: CyHV-2 DNA copies were calculated similarly as described above. The data shown are the mean of triplicates ± SD. (**C**) RT-PCR analysis of total RNA harvested from GCBlat1 cells of passage 32: Lane 1, β-actin; lane 2, *DNA pol* (362 bp); lane 3, *ORF99* (513 bp); M, DNA ladder. One representative experiment of three is shown.

To investigate whether CyHV-2 was present in GCBLat1 cells, total DNA from GCBLat1 cells at passages 32, 46, 51, 52, 60, and 61 were examined by PCR with primers specific to CyHV-2 DNA pol and ORF99, respectively. As shown in Figure 4A, CyHV-2 DNA was detected from all of those tested cells at passage 32, 46, 51, 52, 60, and 61. CyHV-2 DNA copy numbers ranging from 300 to 1000 copies/μg were detected in cells at passage 32, 46, 51, 52, 60, and 61 (Figure 4B). However, no active transcription of *DNA pol* and *ORF99* was observed when total RNA samples from these cells were examined by RT-PCR with primers specific to *DNA pol* or *ORF99*. No virion was observed in these cell pellets under TEM (Data not shown). Therefore, no active CyHV-2 infection was present in these brain-derived primary cells.

### 3.4. CyHV-2 Reactivation upon Chemicals Treatment

**Figure 5:**
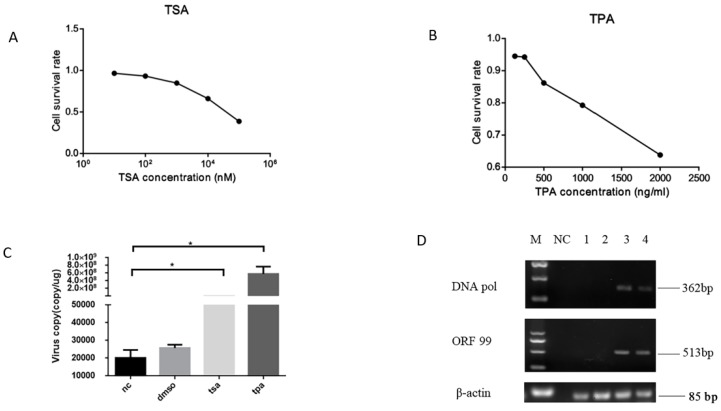
Examination of CyHV-2 DNA and gene transcription in GCBlat1 cells upon chemicals treatment: Cell viabilities of GCBlat1 cells were measured by an MTT assay following treatments with trichostatin A (TSA) (**A**) or phorbol 12-myristate 13-acetate (TPA) (**B**) (80, 40, 20, 10, and 5 μM) or dimethyl sulfoxide (DMSO) for 48 h. (**C**) qPCR analysis of the CyHV-2 DNA from GCBlat1 cells with TSA, TPA, DMSO, or without treatment (nc). CyHV-2 DNA copies were calculated similarly as described above. Data presented as the mean ± SD (*n* = 3). (**D**) RT-PCR analysis of total RNA harvested from treated and untreated GCBlat1 cells: The amplicons of *DNA pol* (362 bp) and *ORF99* (513 bp) are indicated. The amplicons of β-actin gene are used as an internal control to ensure that comparable levels of input RNA were used in RT-PCR. M: DNA ladder; NC: negative control for PCR; lane 1: mock-treated GCBlat1 cells; lane 2: DMSO-treated GCBlat1 cells; lane 3: TPA-treated GCBlat1 cells; lane 4: TSA-treated GCBlat1 cells. One representative experiment of three is shown here. * *p* < 0.05

Chemical TSA or TPA were capable of trigging HSV-1 and other herpesvirus reactivations from latency [21,22]. To determine whether CyHV-2 was latent in GCBLat1 cells, TSA or TPA was included in the GCBLat1 cell culture media. CyHV-2 DNA copies were measured in treated cells and untreated cells. As shown in Figure 5C, 2–8 × 10^8^ viral genome copies/ug were detected in cells treated with TSA and TPA, respectively. Only about 2000–3000 viral genome copies/ug were detected in the untreated and DMSO-treated cells. Viral active gene transcription of *DNA pol* and *ORF99* was observed in GCBLat1 cells treated with TSA or TPA (Figure 5D, lane 3 and 4). The pathogenicity of CyHV-2 released from TPA-treated GCBLat1 cells was subsequently evaluated by experimental infection with apparently healthy gibel carp. Infected fish began to die at five dpi, and the mortality peaked at 7–8 dpi. The clinical signs of diseased gibel carp in this study included gill necrosis, severe skin hemorrhages, and swollen abdomen (data not shown). It suggests infectious virions were produced from CyHV-2 reactivation from latency in GCBLat1 cells.

## 4. Discussion

**Highlights**

> CyHV-2 established latency following primary infection.

> CyHV-2 latency could be reactivated by temperature stress in vivo.

> A novel cell line derived from the brain of gibel carp was established and denoted by GCBLat1.

> The GCBLat1 cell line supports CyHV-2 latency and reactivation.

The above study demonstrated that CyHV-2, like mammalian herpesviruses, can become latent following a primary infection. Importantly, GCBLat1 cells derived from the brain of latently infected gibel carp were also latently infected with CyHV-2. The latently infected GCBLat1 can be reactivated and can produce infectious virions. This cell line offers an in vitro model to investigate the mechanism of latency and reactivation for CyHV-2.

The classic definition of herpesvirus latency is (1) the presence of viral genomes, (2) little or no lytic gene expression, (3) expression of the latency-associated transcripts (LATs), (4) the absence of detectable infectious virion, and (5) ability to be reactivated from latency [23,24]. Although the genes associated with CyHV-2 latency has not been identified in this study, the data presented here meet the characteristic of latent herpesvirus infections. The transcription of two lytic genes of *ORF99* and *DNA pol* were chosen to confirm the lytic infection of CyHV-2. *ORF99* encodes a major envelope protein, and *DNA pol* encodes the DNA polymerase of CyHV-2, which is essential for CyHV-2 multiplication [8,25]. We demonstrated that CyHV-2 genome DNA was detectable without lytic gene transcription in latent fish in vivo (Figure 1D) and latent cell lines in vitro (Figure 4C). However, under temperature stress, viral DNA replication and viral gene transcriptions, such as *ORF99* and *DNA pol*, were detected in fish of 10 months postinfection (Figure 2B,C). These studies confirmed that CyHV-2 became latent in fish exposed to the infection.

Interestingly, GCBLat1 cells derived from CyHV-2 latently infected fish were latently infected also. When the cells were cultured in regular growth media, limited viral genome was detected, but no transcription of CyHV-2 was detectable in GCBLat1 cells (Figure 4). However, when the cells were exposed to Trichostatin A (TSA) or 12-O-tetradecanoyl-13 acetate (TPA), viral DNA replication, gene transcription, and infectious virion production were observed in the tissue cultures. TSA is an inhibitor of histone deacetylases and can trigger HSV-1 activation in vitro [26]. It is believed that TSA can modify the latent viral genome and can convert it from heterochromatin to euchromatin to favor lytic gene expression [27]. TPA, also known as tetradecanoylphorbol acetate, is most commonly used as phorbol ester, binds and activates protein kinase C, and causes a wide range of effects in cells and tissues [28,29]. Both EBV and Kaposi’s sarcoma-associated virus (KHSV) reactivation from latency can be triggered by TPA [30,31]. Here, treatment with TPA could significantly increase the amount of CyHV-2 DNA copies in GCBLat1 cells. It is speculated that TPA can activate the signal transduction enzyme protein kinase C (PKC), which can disrupt the latency phase and favor the reactivation phase [28]. This hypothesis is supported by the observation that TPA induces KHSV reactivation in PEL cell cultures via stimulation of the MAPK/ERK pathway and activation of the early-immediate viral protein RTA, which is important for activation of KHSV latency [32]. In fish, the MAPK ERK1/2, JNK, or p38 pathways were reported to have critical roles in response to hypoxia stress, ammonia, or other environmental factors [33]. Understanding the detailed mechanism of CyHV-2 latency and reactivation would contribute to the prevention and control of CyHV-2 in the aquaculture industry.

Many in vitro cell models were employed to study herpesvirus latency, such as HSV-1 and CMV. For instance, a myeloid progenitor cell line was used to study HCMV latency in vitro [34]. Primary dorsal root ganglion neurons, rat pheochromocytoma cells (PC12), humxan neuroblastoma cells (SH-SY5Y), and lund human mesencephalic neuronal cell line (LUHMES) were indicated to support HSV-1 latency in vitro [10,35]. Although those in vitro systems were capable of supporting the latent-like infection, they require special treatments to establish latency. For example, LUHMES cells require an initial treatment with acyclovir for the first 48 h of infection [35], PC-12 cells need to be differentiated with nerve growth factor (NGF) first in order to support HSV-1 latent infection [22]. Lastly, many of those cells are not derived from the natural host and may not simulate the real host–virus infection during latency [35]. GCBLat1, derived from natural host gibel carp with CyHV-2 latency, could maintain CyHV-2 latency without any additional treatment. To our knowledge, this is the first description of a cell line that can support fish herpesvirus latency and that can be reactivated following treatments. GCBLat1 cell line offers a useful platform for molecular and biochemical analyses of CyHV-2 latency and reactivation.

In conclusion, CyHV-2, like many herpesviruses, can become latent and reactivated from latent infection. GCBLat1 cell line is latently infected with CyHV-2 and CyHV-2 latency can be reactivated in vitro. GCBLat1 cell line have potential to serve as an in vitro model to study molecular aspects of CyHV-2 latency and reactivation.

## Figures and Tables

**Table 1:** Primer pairs used to detect CyHV-2 DNA and gene transcription.

Name^a^	Target	Prime Sequence
DNA pol-F	DNA polymerase	5′-CCCAGCAACATGTGCGACGG-3′
DNA pol-R	DNA polymerase	5′-CCGTARTGAGAGTTGGCGCA-3′
ORF99-F	ORF99	5′-GTTACCCGAAGATACCCAT-3′
ORF99-R	ORF99	5′-TGCCGTTAGAAGGAGAAG-3′
actin-F	β-actin	5′-GCTATGTGGCTCTTGACTTCGA-3′
actin-R	β-actin	5′-CCGTCAGGCAGCTCATAGCT-3′
qpol-F	DNA polymerase	5′-GAAGGGCGGTAAAGTGTT-3′
qpol-R	DNA polymerase	5′-TCGCTGTGCGGGTATT-3′

^a^ Forward and reverse primers are indicated by F and R, respectively, in the primer names.

## References

[1] Hedrick R.P., Waltzek T.B., Mcdowell T.S. (2006). Susceptibility of Koi Carp, Common Carp, Goldfish, and Goldfish × Common Carp Hybrids to Cyprinid Herpesvirus2 and Herpesvirus3. J. Aquat. Anim. Health.

[2] Daněk T., Kalous L., Vesel T., Krásová E., Knytl M. (2012). Massive mortality of Prussian carp Carassius gibelio in the upper Elbe basin associated with herpesviral hematopoietic necrosis (CyHV-2). Dis. Aquat. Org..

[3] Fichi G., Cardeti G., Cocumelli C., Vendramin N., Susini F. (2013). Detection of Cyprinid herpesvirus 2 in association with an Aeromonas sobria infection of Carassius carassius (L.), in Italy. J. Fish Dis..

[4] Groff J.M., LaPatra S.E., Munn R.J., Zinkl J.G. (1998). A Viral Epizootic in Cultured Populations of Juvenile Goldfish Due to a Putative Herpesvirus Etiology. J. Vet. Diagn. Investig..

[5] Adamek M., Hellmann J., Jung-Schroers V., Teitge F., Steinhagen D. (2018). CyHV-2 transmission in traded goldfish stocks in Germany-A case study. J. Fish Dis..

[6] Ito T., Maeno Y. (2014). Effects of experimentally induced infections of goldfish Carassius auratus with cyprinid herpesvirus 2 (CyHV-2) at various water temperatures. Dis. Aquat. Org..

[7] Wei C., Iida H., Chuan Q., Tanaka M., Kato G., Sano M. (2019). Persistence of cyprinid herpesvirus 2 in asymptomatic goldfish *Carassius auratus* (L.) that survived an experimental infection. J. Fish Dis..

[8] Davison A.J., Kurobe T., Gatherer D., Cunningham C., Korf I., Fukuda H., Hedrick R.P., Waltzek T.B. (2013). Comparative Genomics of Carp Herpesviruses. J. Virol..

[9] Grinde B. (2013). Herpesviruses: Latency and reactivation—Viral strategies and host response. J. Oral Microbiol..

[10] Nikki M.T., Steven J.T. (2017). Herpes simplex virus establishment, maintenance, and reactivation: In vitro modeling of latency. Pathogens.

[11] Lisa L.D., Lori D.K., David A.T. (1996). Detection of the latent form of Epstein-Barr virus DNA in the Peripheral blood of healthy individuals. J. Virol..

[12] Eide K.E., Miller-Morgan T., Heidel J.R., Kent M.L., Bildfell R.J., LaPatra S., Watson G., Jin L. (2011). Investigation of Koi Herpesvirus Latency in Koi. J. Virol..

[13] Reed A.N., Izume S., Dolan B.P., LaPatra S., Kent M., Dong J., Jin L. (2014). Identification of B Cells as a Major Site for Cyprinid Herpesvirus 3 Latency. J. Virol..

[14] Virgin H.W., Wherry E.J., Ahmed R. (2009). Redefining Chronic Viral Infection. Cell.

[15] Aneja K.K., Yuan Y. (2017). Reactivation and Lytic Replication of Kaposi’s Sarcoma-Associated Herpesvirus: An Update. Front. Microbiol..

[16] Lin L., Chen S., Russel D.S., Löhr C.V., Milston-Clements R., Song T., Miller-Morgan T., Jin L. (2017). Analysis of stress factors associated with KHV reactivation and pathological effects from KHV reactivation. Virus Res..

[17] Luo Y.Z., Lin L., Liu Y., Wu Z.X., Yuan J.F. (2013). Hematopoietic necrosis of cultured Prussian carp, Carassius gibelio (Bloch), associated with Cyprinid herpesvirus 2. J. Fish Dis..

[18] Ito T., Kurita J., Ozaki A., Sano M., Ototake M. (2013). Growth of Cyprinid herpesvirus 2 (CyHV-2) in cell culture and experimental infection of goldfish Carassius auratus. Dis. Aquat. Org..

[19] Gui J., Liang S., Zhu L., Jiang Y. (1993). Discover of Multiple Tetraplolds in Artificially Propagated Populations of Allogynogenetic Silver Crucian Carp and Their Breeding Potentials. Chin. Sci. Bull..

[20] Lua J., Xu D., Lu L. (2018). A novel cell line established from caudal fin tissue of Carassius auratus gibelio is susceptible to cyprinid herpesvirus 2 infection with the induction of apoptosis. Virus Res..

[21] Danaher R.J., Jacob R.J., Steine M.R., Allen W.R., Hill J.M., Miller C.S. (2005). Histone deacetylase inhibitors induce reactivation of herpes simplex virus type 1 in a latency-associated transcript (LAT)-independent manner in neuronal cells. J. Neurovirol..

[22] Danaher R.J., Jacob R.J., Miller C.S. (1999). Establishment of a quiescent herpes simplex virus type 1 infection in neurally-differentiated PC12 cells. J. Neurovirol..

[23] Reed A., Lin L., Ostertag-Hill C., Wang Q., Wu Z., Miller-Morgan T., Jin L. (2017). Detection of ORF6 protein associated with latent KHV infection. Virology.

[24] Roizman B., Sears A.E. (1987). An Inquiry into the Mechanisms of Herpes Simplex Virus Latency. Annu. Rev. Microbiol..

[25] Li L., Luo Y., Gao Z., Huang J., Zheng X., Nie H., Zhang J., Lin L., Yuan J. (2015). Molecular characterisation and prevalence of a new genotype of Cyprinid herpesvirus 2 in mainland China. Can. J. Microbiol..

[26] Arthur J.L., Scarpini C.G., Connor V., Lachmann R.H., Tolkovsky A.M., Efstathiou S. (2001). Herpes simplex virus type 1 promoter activity during latency establishment, maintenance, and reactivation in primary dorsal root neurons in vitro. J. Virol..

[27] Nehme Z., Pasquereau S., Herbein G. (2019). Control of viral infections by epigenetic-targeted therapy. Clin. Epigenet..

[28] Ye F., Zhou F., Bedolla R.G., Jones T., Lei X., Kang T., Guadalupe M., Gao S.-J., Früh K. (2011). Reactive Oxygen Species Hydrogen Peroxide Mediates Kaposi‘s Sarcoma-Associated Herpesvirus Reactivation from Latency. PLoS Pathog..

[29] Rebois R.V., Patel J. (1985). Phorbol ester causes desensitization of gonadotropin-responsive adenylate cyclase in a murine Leydig tumor cell line. J. Biol. Chem..

[30] Tobias S., Heiko A. (2018). “Novel” Triggers of Herpesvirus Reactivation and Their Potential Health Relevance. Front. Microbiol..

[31] Murata T. (2014). Regulation of Epstein-Barr virus reactivation from latency. Microbiol. Immunol..

[32] Cohen A. (2006). An essential role of ERK signalling in TPA-induced reactivation of Kaposi’s sarcoma-associated herpesvirus. J. Gen. Virol..

[33] Johnson G.L., Lapadat R. (2002). Mitogen-Activated Protein Kinase Pathways Mediated by ERK, JNK, and p38 Protein Kinases. Science.

[34] O’Connor C.M., Murphy E.A. (2012). A myeloid progenitor cell line capable of supporting human cytomegalovirus latency and reactivation, resulting in infectious progeny. J. Virol..

[35] Edwards T.G., Bloom D.C. (2019). Lund human mesencephalic (LUHMES) neuronal cell line supports herpes simplex virus 1 latency in vitro. J. Virol..

